# A 10-hour within-participant magnetoencephalography narrative dataset to test models of language comprehension

**DOI:** 10.1038/s41597-022-01382-7

**Published:** 2022-06-08

**Authors:** Kristijan Armeni, Umut Güçlü, Marcel van Gerven, Jan-Mathijs Schoffelen

**Affiliations:** Donders Institute for Brain, Cognition and Behaviour, Radboud University, Nijmegen, The Netherlands

**Keywords:** Language, Neural encoding

## Abstract

Recently, cognitive neuroscientists have increasingly studied the brain responses to narratives. At the same time, we are witnessing exciting developments in natural language processing where large-scale neural network models can be used to instantiate cognitive hypotheses in narrative processing. Yet, they learn from text alone and we lack ways of incorporating biological constraints during training. To mitigate this gap, we provide a narrative comprehension magnetoencephalography (MEG) data resource that can be used to train neural network models directly on brain data. We recorded from 3 participants, 10 separate recording hour-long sessions each, while they listened to audiobooks in English. After story listening, participants answered short questions about their experience. To minimize head movement, the participants wore MEG-compatible head casts, which immobilized their head position during recording. We report a basic evoked-response analysis showing that the responses accurately localize to primary auditory areas. The responses are robust and conserved across 10 sessions for every participant. We also provide usage notes and briefly outline possible future uses of the resource.

## Abstract (summary)

**Table:** 

Measurement(s)	brain activity measurement
Technology Type(s)	Magnetoencephalography
Factor Type(s)	auditory story
Sample Characteristic - Organism	Homo sapien

## Background & Summary

Conveying and understanding the world through narratives is a pervasive and fundamental aspect of the human culture^1^. The storytelling and sense-seeking propensity of the brain allows us to relate the temporally varying and fleeting nature of the world into a stable and meaningful chain of events, entities and their relations^2,3^. In recent years, cognitive neuroscientists of language have seen an increased interest in studying the brain responses when humans read or listen to narratives^4–10^. Narratives afford the study of brain responses to language input which is highly contextualized and contains the rich variety of temporal dynamics and representations (e.g. phonemic, lexical, phrasal, morphemic, sentential)^11^. Given this inherent richness of neuronal dynamics, model-based investigations — where cognitive hypotheses on multiple levels are operationalized and tested simultaneously^12^ — are particularly suitable in narrative neuroscience.

It is not a coincidence then that narrative cognitive neuroscience has been encouraged by the simultaneous development and availability of novel modeling techniques in the fields of natural language processing (NLP) and computational linguistics^6,13^. Echoing the recent revival of research in artificial neural networks (ANNs) and deep learning^14,15^, NLP has seen considerable progress in research applications using ANN architectures^16^. Earlier breakthroughs were achieved by the development of the so-called neural language models, that is, ANNs optimized to solve the next-word prediction task^17,18^. This development was further underscored by the finding that the numeric vector word representations, derived by using such architectures, develop human-interpretable structure. For example, it is well-established that the numeric word vectors can reflect lexical similarity of the encoded words^19,20^. Most recently, NLP has witnessed a trend towards pretraining generic large-scale neural language models on unprecedented amounts of raw text and successfully using the pretrained models for improved performance on downstream language tasks^21–23^.

The availability of language models that can process connected text has increased the scope of cognitive neuroscientists’ toolkit for probing the relationship between computational language representations and the neural signals. Mirroring the successes in computer vision^24^ and the subsequent modeling of neural processing in visual perceptual hierarchies^25–27^, computational linguists are beginning to interpret *how* language models achieve their task performance^28–30^ and what is the correspondence between such pretrained model representations and neural responses recorded when participants engage in similar language tasks^31–37^. On the one hand, task-optimized ANNs therefore serve as a tool and a framework that allow us to operationalize and identify which computational primitives serve as the candidate hypotheses for explaining neural data^38–41^. On the other hand, data from neuroscience can provide important biological constraints for the ANN-based processing architectures^42–44^.

Yet, in order to incorporate neurophysiological constraints in the training of brain-based ANNs, we need sufficiently large and high-quality language-brain datasets and resources that allow direct training and testing of such complex models^43^. Neural networks are known to be ‘data-hungry’. That is, because the number of optimized parameters is typically very large, the models need to be constrained by sufficient amounts of data points in order to reduce the error variance during training^45^. In studies where the cognitive hypothesis of interest is embodied in the experimental design, the nature of well-controlled hand-crafted stimuli typically puts a limit to the number of available train-test samples *within each participant*. For example, the dataset from the landmark study by^31^ contains fMRI data for a total of 60 nouns. Since then, the availability of model-based analyses approaches^13^ has led to increased curation and sharing of dedicated language neuroimaging datasets that leverage larger amount of data points *across* large numbers of participants^46,47^ It is known that the increasing amount of training repetitions *within each participant* improves predictive performance of models. This was shown, for example, in predicting visually-evoked MEG responses^48^ and in speech/text synthesis on the basis of intracranial electrophysiology recordings^49,50^. Given the recent interest in the interpretability of ANN language models^28^ and the development of ANN models of brain function^51^, care must be taken to ensure that the to-be investigated models trained on neural data of individual participants are reliably estimated to begin with^52^.

**Fig. 1:**
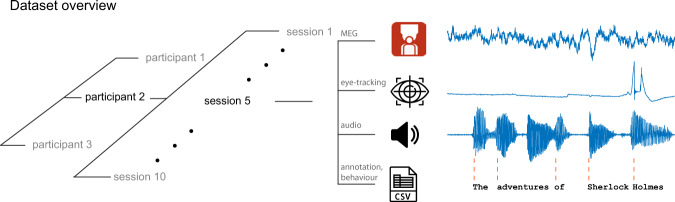
The structure of the dataset. We recorded the MEG from 3 participants, 10 separate recording sessions each. In each session, we recorded MEG data (275-channel axial gradiometer CTF system) while participants listened to audiobooks (The Adventures of Sherlock Holmes) in English. Along with the MEG data, we also tracked eye-movements and pupil dilations. After story listening in each session, participants answered short behavioral questionnaires about their narrative comprehension experience. We also provide the timings of word onsets for every story in the dataset that can be used to relate pretrained models (or other linguistic features) to the MEG data. The icons in the figures from https://www.flaticon.com/authors/freepik.

Here we describe a narrative comprehension magnetoencephalography (MEG) data resource recorded while three participants listened nearly 10 hours of audiobooks each (see Fig. 1). MEG is a non-invasive technique for measuring magnetic fields induced by synaptic and transmembrane electric currents in large populations (∼10,000–50,000 cells) of nearby neurons in the neocortex^53,54^. Due to the rapid nature of electrophysiological responses and the millisecond sampling rate of the MEG hardware^55,56^, it is frequently the method of choice for studying the neural basis of cognitive processes related to language comprehension. The target applications of this dataset are studies aiming to build and test novel encoding or decoding models^57^ of spoken narrative comprehension (for English), to evaluate theories of narrative comprehension at various timescales (e.g. words, sentences, story), and to test current natural language processing models against brain data.

## Methods

This report follows the established conventions for reporting MEG data^58,59^.

### Participants

A total of 3 (1 female) aged 35, 30, and 28 years were included in the study. All three participants were native English speakers (UK, US and South African English). All participants were right-handed and had normal or corrected-to-normal vision. The participants reported no history of neurological, developmental or language deficits. In the written informed consent procedure, they explicitly consented for the anonymized collected data to be used for research purposes by other researchers. The study was approved by the local ethics committee (CMO — the local “Committee on Research Involving Human Subjects” in the Arnhem-Nijmegen region) and followed guidelines of the Helsinki declaration.

Our participants were speakers of three distinct English dialects (UK, US and South African English). While this could be a potential source of inter-participant variability in neural responses, the nature of out-group accent effects in M/EEG remains debated^60^. Importantly, we speculate that accent-driven variability is less of an issue for the current dataset where two participants listened to an out-group dialect (British English) where the lack of exposure, we reason, was likely not critical.

Finally, it is worth pointing out, given the emphasis on the number of data points *within* each participant, that this design decision introduces a trade-off in terms of what kind of conclusions and inference can be achieved. Specifically, given the small number of participants, the current dataset is not well suited for *group-level inference*. In other words, if the goal of a potential analysis is to generalize a phenomenon across participants, the current data resource should best be complemented by another resource that permits group-level inference. However, there is opportunity even for studies that primarily aim to achieve group-level generalization — namely, the large number of recordings within the same participant minimize the sources of variability which makes this resource a valuable starting point for exploratory analyses. Such data-driven, exploratory analyses can lead to concrete hypotheses which can then be tested in a confirmatory study, for example, on a dataset with a larger number of participants.

### Stimulus materials

We used The Adventures of Sherlock Holmes by Arthur Conan Doyle as read by David Clarke and distributed through the LibriVox public library (https://librivox.org). The primary considerations in the selection of these stimulus materials were: a) the expectation of a relatively restricted or controlled vocabulary limited to real-life story contents (as opposed to in, for example, highly innovative styles of writing or fantasy literature where the dimensionality of semantic spaces can be expected to be higher), which made it reasonable to expect that models would be able to meaningfully capture the text statistics, b) sufficient number of books which are available as plain text, and c) the availability of corresponding audiobooks (the plain text of The Adventures of Sherlock Holmes was obtained from https://sherlock-holm.es/stories/plain-text/advs.txt, accessed on September 11, 2018)

Each individual story was further divided into subsections. The subsections were determined by us after reading through the stories. We made sure that the breaks occurred in meaningful text locations, for example, that prominent narrative events are not split across two runs. Stimuli and materials are available in the ‘/stimuli’ folder in the top-level directory. The full specification of stimuus materials is available in Table 1 of the supplementary materials.

**Table 1:** P-values for comparisons in the canonical correlation analysis (CCA) of repeated segments (*cca*) and randomly selected segments (*cca*^′^).

participant comparison	sub-001	sub-002	sub-003
^*cca*^*within* ^vs. *cca*^*across*	0.37	0.11	0.77
^*cca*^*’within* ^vs. *cca*^*’across*	0.36	0.19	0.94
*cca*_*within*_ vs. *cca*^’^_*within*_	< 0.001	0.001	< 0.001
^*cca*^*across* ^vs. *cca*^*’across*	< 0.001	0.0018	< 0.001

The ‘within’ and ‘across’ subscripts denote CCA performed on paired runs selected within the same recording session and runs selected across different recording sessions, respectively.

### Word timing information

To determine word onsets and offsets in every auditory story we performed automatic forced alignment of the audio recordings and the text obtained from Project Gutenberg. We used the Penn Forced Aligner toolkit^61^ with pretrained acoustic models for English. Tokenization of the story text was performed with the tokenizer module in the Spacy natural language processing library (https://spacy.io/api/tokenizer).

Some of the tokenization rules will result in tokens that do not lend themselves well as a unit of analysis for the corresponding acoustic forms. Notably, the tokenizer by default will split contractions: (“don’t” –> “do” “not”). To be able to use contracted forms as inputs to the forced alignment algorithm, we post-edited the split contractions back to the original form. Word tokens not included in the precompiled model dictionary were added manually upon inspection of the forced alignment log files (e.g. proper names, see the “dict.local” file for the full list). The arpabet pronunciation codes for missed tokens were generated using the web interface of the CMU LOGIOS Lexicon Tool (http://www.speech.cs.cmu.edu/tools/lextool.html). Symbols for stress in pronunciations were added manually to the generated arpabet pronunciation codes.

### Task and experimental design

**Fig. 2:**
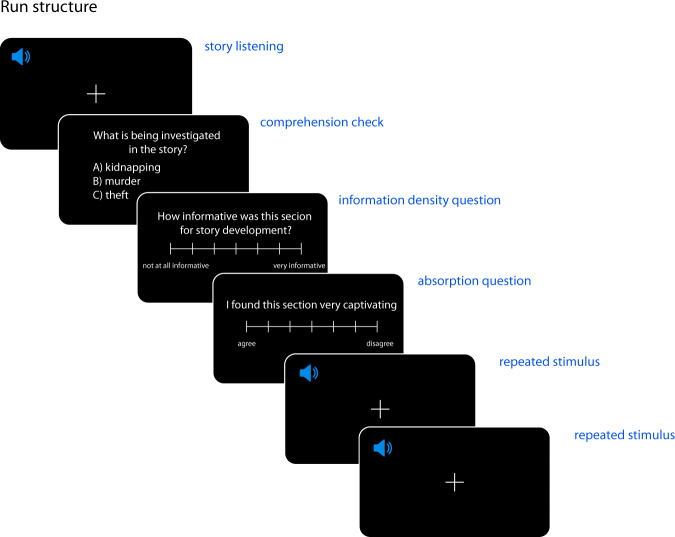
Trial structure. In each session, the participants listened to one story from the collection ‘The Adventures of Sherlock Holmes’. Each story was further split into subsections which correspond to experimental runs. The structure of one run is depicted here. After listening to the subsection, the participants answered a simple multiple choice comprehension question and rated the information density and absorption levels of the heard subsection. After the responses, each participants listened to a short story snippet repeated twice. For the repeated section, the same stimulus (taken from Sherlock Holmes story not used in the main set) was used after each run. The speaker icon created by Pixel Perfect (https://www.flaticon.com/authors/pixel-perfect).

Each of the 3 participants listened to the 10 stories from the Adventures of Sherlock Holmes. A separate MEG session consisted of listening to a single story from the collection. Each recording session took place on a separate day. A single run (i.e. an uninterrupted period of continuous data acquisition) consisted of participants listening to a subsection of a story (see Fig. 2). The order of story and run presentation were kept the same for all participants (see Table 1, supplementary information). Participants were instructed to listen attentively for comprehension. After each run, the participants answered comprehension questionnaires and reported their literary experience and were able to take short breaks. The experimenter was available for clarification prior to the beginning of the recording.

Each story was presented binaurally via a sound pressure transducer through two plastic tubes terminating in plastic insert earpieces. A black screen with a fixation cross was maintained while participants listened to the stories. Presentation of the auditory stories was controlled with Presentation software (version v 16.4., build 06.07.13, NeuroBehavioral Systems Inc.).

### Comprehension check

Comprehension check was used after each run to make sure participants were following the story contents. Each comprehension check consisted of 1 multiple choice question per run with 3 possible answers. The questions were designed by us and should have been possible to answer correctly for people who had read the stories with normal attention (example question: ‘What is being investigated in the story?’). The participants indicated their response by means of a button box and had no time limit to do so. For the full questionnaire with answers see ‘questions_tabular.txt’.

### Information density and absorption questions

After each run, the participants reported their perceived informativeness of the heard story subsection (by answering the question: ‘How informative do you think this section was for the story development?’) and indicated their level of absorption (i.e. level of agreement with the statement: ‘I found this section very captivating’). They indicated their perceived information density by rating their response on a visual scale from 1 (‘Not at all informative’) to 7 (‘Very informative’). They indicated their level of absorption by rating their response on a visual scale from 1 (‘Disagree’) to 7 (‘Agree’).

### Literary appreciation

At the end of each recording session, the participants were asked to report appreciation of the heard story. The appreciation questionnaire was the one used by^62^ who adapted the version by^63^. The questionnaire consisted of a general score of story liking (*I thought this was a good story*.), thirteen statements with adjectives that indicated their impression of the story (e.g. *I thought this story was*... {*Beautiful*... *Entertaining*, *Ominous*}). Finally, they rated their agreement to 6 statements regarding their enjoyment of the story (adapted from^64^; e.g. *I was constantly curious about how the story would end*). Participants rated their story liking (statements with adjectives), and the statements regarding their enjoyment on a 7-point scale ranging from 1 (‘Disagree’) to 7 (‘Agree’). For the full questionnaire see Table 6.

### MEG data acquisition

**Fig. 3:**
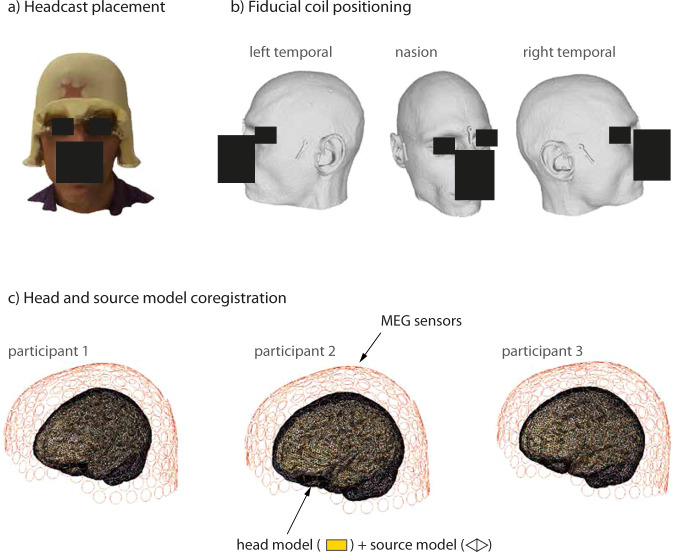
(**a**) Example of headcast placement on a participants’ head. (**b**) Example of the geometrical model of the head and fiducial coil positioning. This headshape model was used for head cast production. (**c**) The outcome of the coregistration procedure, shown are head and source models relative to MEG sensors.

We recorded MEG data (275-channel axial gradiometer CTF system) while participants listened to audiobooks in English in a magnetically shielded room. The MEG signals were digitized at a sampling rate of 1200 Hz (the cutoff frequency of the analog anti-aliasing low pass filter was 300 Hz). Throughout the recording, the participants wore an MEG-compatible headcast^65^ (see Fig. 3). Per convention, three head localizer coils were attached to the inner side of the headcast at the nasion, left pre-auricular, and right pre-auricular sites. Head position was monitored during the recording using a custom build monitoring tool^66^.

#### Empty room recordings

**Fig. 4:**
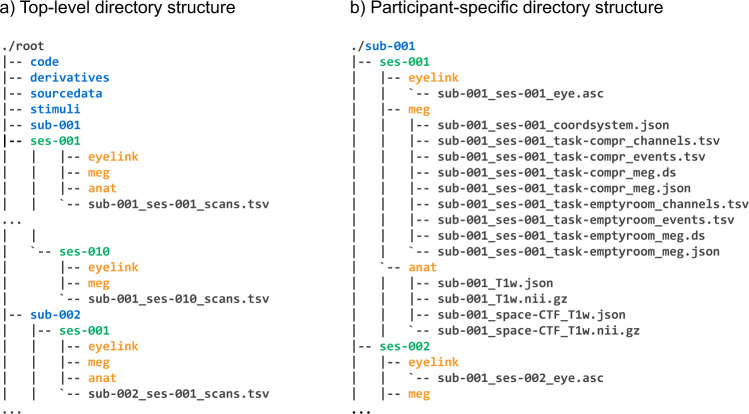
Data records overview.

Immediately before or after each recording session (depending on lab availability), we performed empty room recordings. These recordings lasted for approximately 5 minutes. Empty room recordings are located in a separate .ds folder in the respective session folder (see Fig. 4, panel b).

#### Repeated stimulus recordings

In between runs, we recorded MEG responses to a short (half minute) excerpt from The Adventures of Sherlock Holmes which was not used during the main task (‘noise_ceiling.wav’). The stimulus was repeated twice between runs. The MEG and Presentation trigger codes marking the onset and offset of the repeated stimuli have the values 100 and 150, respectively.

### MRI data acquisition

To produce the headcast, we needed to obtain accurate images of the participants’ scalp surface. To this end, we obtained structural MRI scans with a 3T MAGNETOM Skyra MR scanner (Siemens AG) at the Donders Centre for Cognitive Neuroimaging in Nijmegen, the Netherlands. During the scanning procedure, the participants lay in the supine position with a vitamin E capsule attached to their right ear as a marker for image orientation. We used a fast low angle shot (FAST) sequence with the following image acquisition parameters: slice thickness of 1 mm; field-of-view of 256 × 256 × 208 mm along the phase, read, and partition directions respectively; echo time of 1.59 msec; time to repeat (TR) was set to 4.5 msec. The readout bandwidth was 510 Hz per pixel. The acquisition time was 2 min 23 sec.

### MRI-MEG coregistration

To co-register the structural MRI images to the MEG coordinate space, we first transformed the individual participant’s MRI images from the voxel coordinate system to the ACPC head coordinate system by placing the origin of the head coordinate system to the anterior commissure as interactively identified on the structural brain images (see http://www.fieldtriptoolbox.org/faq/anterior_commissure/). We then used the mesh describing the participants’ head shape (see Fig. 3, panel b) and extracted from it the meshes corresponding to the nasion, left pre-auricular, and right pre-auricular fiducial coils. Once the meshes describing the coil geometry were extracted from the head shape mesh, we localized the center points of the each of the three coils. These center points of the coils were taken to represent the locations where the fiducial coils were placed during the recordings (as the coils were actually placed in the empty slots at the positions in the geometric model). These extracted coordinate points were then manually inspected and appropriately defined as the nasion, the left pre-auricular and right pre-auricular points based on the signs and values of their x, y, and z coordinates. The above procedure allowed us to coregister the MRI image to the MEG coordinate space. The outcome of the coregistration for each participant is shown in Fig. 3.

### Eye-tracking data acquisition

Concurrently with the MEG, we recorded participants’ eye-movements. We used the Eyelink 1000 Eyetracker (SR Research ©) at a sampling rate of 1000 Hz. The 9-point scheme was used for calibration after positioning the participant within the MEG dewar and prior to starting the MEG data acquisition. The participant’s left eye was tracked in all cases.

## Data Records

The dataset can be accessed at the data repository of the Donders Institute for Brain, Cognition and Behaviour^67^. The dataset is shared under the Data use agreement for identifiable human data (version RU-DI-HD−1.0, https://data.donders.ru.nl/doc/dua/RU-DI-HD-1.0.html?3), developed by the Donders Institute and Radboud University, which specifies the conditions and restrictions under which the data is shared. The dataset organization follows a BIDS-like specification for storing and sharing the MEG data^68^. The organization of the dataset directory is presented in Fig. 4. The three folders at the highest level (Fig. 4a) contain the data for three participants (*sub-001*, *sub-002*, and *sub-003*). Each participant directory contains data folders for respective sessions (*ses-001*, *ses-002* etc.) with subfolder for each respective data modality (*eyelink* for eye-tracking data, *meg* for MEG data etc.). The organization of session-specific directories is displayed in panel B of Fig. 4. The contents of the individual folders in the dataset directory are briefly described below.

### Code (‘/code’)

The code folder contains source MATLAB code for the analyses presented in this report and additional wrapper scripts that – together with shared preprocessing data – represent minimal working examples of the present analysis pipeline. The code base relies heavily on the routines of the FieldTrip toolbox^69^.

The high level ‘/code’ folder contains two subfolders. The ‘/pipeline’ subfolder contains the MATLAB scripts and functions that were used in preprocessing and the main analyses. The code is further grouped into ‘audio’, ‘meg’, ‘models’, and ‘utilities’ folders each containing scripts and functions for the respective parts of preprocessing steps. The ‘/plots’ folder contains the two scripts that were use to plot the figures for technical validation below. Further information about the code usage is provided in the ‘README.txt’ files in ‘code/pipeline/meg’ and ‘code/plots’ folders.

### Stimulus folder (‘/stimuli’)

The ‘/stimuli’ folder contains the .wav audio files used in the recordings and the corresponding text files (see Stimulus Materials). The naming of the files follows the ‘%02d_%d’ format where the first digit (with a front-padded zero digit) marks the session and the second digit (not zero padded) codes the run number in that session (see Table 1 in Supplementary materials). In addition, the ‘/stimuli’ folder also contains the input (‘tokens.txt’) and the output (‘pfa.txt’) of the forced alignment (see Section ‘Word Timing Information’) providing word onset timings for all the words in the input tokens lists. The timing information in the ‘pfa.txt’ files follows the TextGrid object syntax of the Praat software (http://www.fon.hum.uva.nl/praat/manual/TextGrid.html).

### Presentation log files and responses (‘/sourcedata/responses’)

The ‘/sourcedata’ folder contains two subdirectories. The ‘logfiles’ subdirectory contains the log files generated by the Presentation scripts (‘*ĺog’). The ‘responses’ subdirectory contains the participants’ responses to the comprehension check, their absorption scores and density scores after each run (‘*_beh.txt’). It also contains the responses to the appreciation questionnaire (‘*_appreciation.txt’) at the end of the session.

### Derivatives folder (‘/derivatives’)

The ‘/derivatives’ folder contains the outputs of specific preprocessing steps related to anatomical and MEG data used in the technical validation presently and that can be potentially of use in further analysis pipelines.

#### Anatomical atlas (‘./atlas‘)

The ‘atlas’ subfolder contains the anatomical parcellation of cortical source points into brain areas or parcels (see Section ‘Beamformer’ below for further details). The FieldTrip MATLAB stucture (see https://github.com/fieldtrip/fieldtrip/blob/release/utilities/ft_datatype_parcellation.m) defining the surface-based descriptions is provided in the corresponding ‘*.mat’ files. Files containing the anatomical labels are shared in the GIFTI file format (‘*label.gii’, see https://www.humanconnectome.org/software/workbench-command/-gifti-help). The cortical parcellations are provided at 3 different resolutions (32k, 8k,and 4k source points per hemisphere). The analyses in this report are based on the 8k resolution parcellation scheme. Finally, we provide two inflated cortical surface descriptions that were used for visualization purposes presently (‘cortex_inflated.mat’ and ‘cortex_inflated_shifted.mat’).

#### Anatomical preprocessing (‘./fieldtrip-anatomy’)

The ‘./fieldtrip-anatomy’ folder contains, for each participant, the data used in source reconstruction (see Section ‘Beamformer’ in ‘Technical validation’ below). That is, it contains the volume conduction model (‘*_headmodel.mat’), the forward projection matrices (‘*_leadfield.mat’), and the description of source locations (‘*_sourcemodel.mat’). It additionally contains the transformation matrices that can be used to transform geometrical objects between the MNI, the ACPC, and the CTF coordinate systems (‘*_transform_*.mat’, for more information on coordinate systems see https://www.fieldtriptoolbox.org/faq/coordsys/.

#### Preprocessing (‘./fieldtrip-preprocessing’)

The ‘./fieldtrip-preprocessing’ folder contains MATLAB data structures containing information from various stages of pre-processing. Specifically, for each participant and each recording session, it provides the channel selection (‘chansel.mat’), trial definition – that is, story onset and offsets expressed in sample points – (‘trl.mat’), squid and muscle artifact definitions (‘*_squid.mat’ and ‘*_muscle.mat’, respectively), the component mixing and unimixing matrices (‘comp.mat’), the selected components for eye-blink component removals (‘selcomp.mat’), and the audio delay between the audio recording onset in each session and the MEG system trigger sending (‘audiodelay.mat’). These data structures are computed separately for the story-listening runs and the repeated stimulus runs (see Section Repeated stimulus recordings).

#### Cortical surfaces (‘./workbench-anatomy’)

The ‘./workbench-anatomy’ folder contains the surface-registered cortical surface models in the GIFTI file format (‘**.gii’), as generated from the Freesurfer output by the hcp-workbench tool (see ‘Cortical sheet reconstruction’ below for more information).

### Raw MEG data folder (‘/sub-00X/ses-00Y/meg’)

The ‘/meg’ folder contains two raw MEG ‘.ds’ datasets; the task-based narrative comprehension (with the infix ‘task-compr’) and the session-specific empty room recording (‘task-empty’). In addition, the folder contains several BIDS sidecar files with meta information about the datasets: a ‘.json’ file with MEG acquisition parameters (‘*_meg.json’), tab-separated table with MEG channel information description (‘*_channels.tsv’), and a table containing detailed timing information about relevant events that occurred during the measurements (‘*_events.tsv’), specifically word and phoneme onset times as obtained from the forced alignment procedure (see Table 4).

### Eye-tracking data (‘/sub-00X/ses-00Y/eyelink’)

The folder contains Eyelink 1000 Eyetracker data converted into the ‘ascii’ (‘*.asc’) format. Note that the eye-tracking information (eye movements and pupil dilation) was also saved as separate data channels in the MEG datasets (see Table 5).

### MRI data (‘sub-00X/ses-001/anat’)

The folder contains anonymized structural MRI images (nifti file format). The ‘/anat’ folder is only present in the first session folder (‘ses-001’) of every participant.

## Technical Validation

All participants were monitored during data acquisition to ensure task compliance and general data quality. As a technical validation, we perform the analysis of the amount of head movement for each of the three participants and compare it to the dataset recorded without headcasts. We also perform a basic auditory evoked-response analysis and source localization for every participant and every session.

### Head movement

In a previous study, Meyer *et al*.^65^ have shown that it was possible to reposition the absolute head position to 0.6 SD across 10 repositionings of the participant wearing a headcast. Here, we report the displacement in the x (left-right), y (left-right), and z (up-down) directions of the circumference of the nasion and the left and right coils. We extracted the head coil localization (‘HLC*’) channels and epoched the session-specific dataset into trials of 1 minute length. We first computed the mean absolute displacement in x, y, and z directions across each 1-minute trial. We then computed the circumcenter of the nasion and the left and right coils in the x, y, and z dimensions per trial. This resulted in a measure of head position and orientation in x, y and z coordinates per trial. Finally, we centered the obtained head position values across the trial dimension by subtracting the mean head position in the specific direction.

**Fig. 5:**
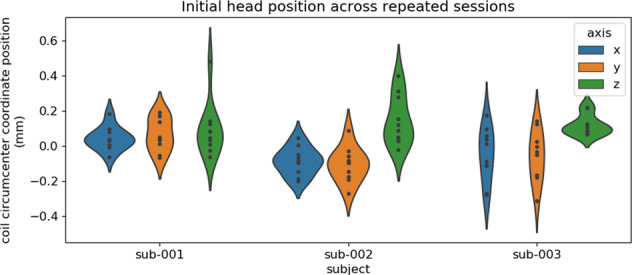
Session-initial head displacement per participant in all 10 sessions.

In Fig. 5, we report the average head position displacement across the first minute of recording. The figure shows that head positioning at the onset of a new session was achieved within 1 millimeter accuracy and for the most part within 0.5 millimeters which is in line with previous reports^65^.

**Fig. 6:**
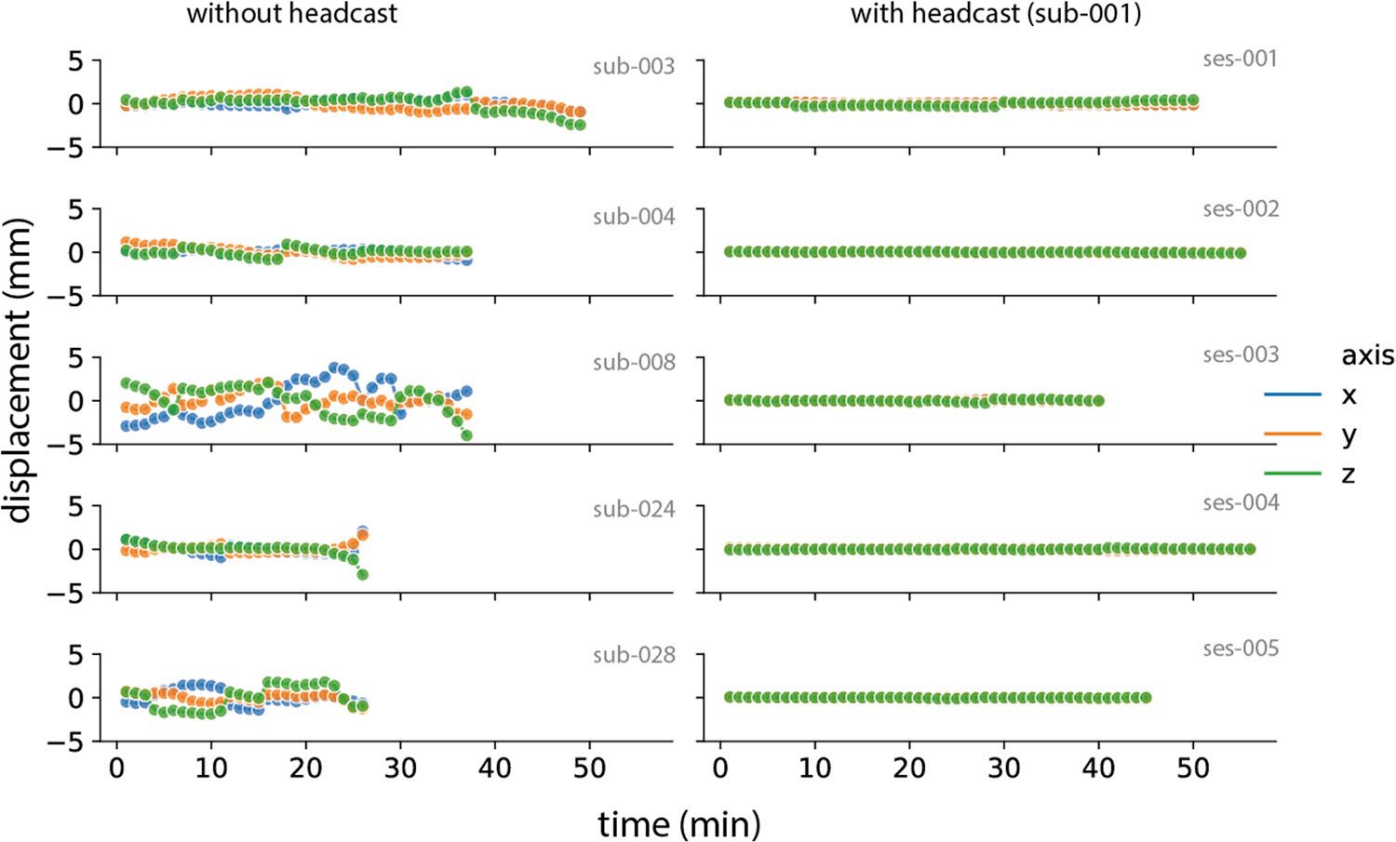
Comparison of head movement for five sessions in a dataset without headcast (left) and the current dataset (right).

**Fig. 7:**
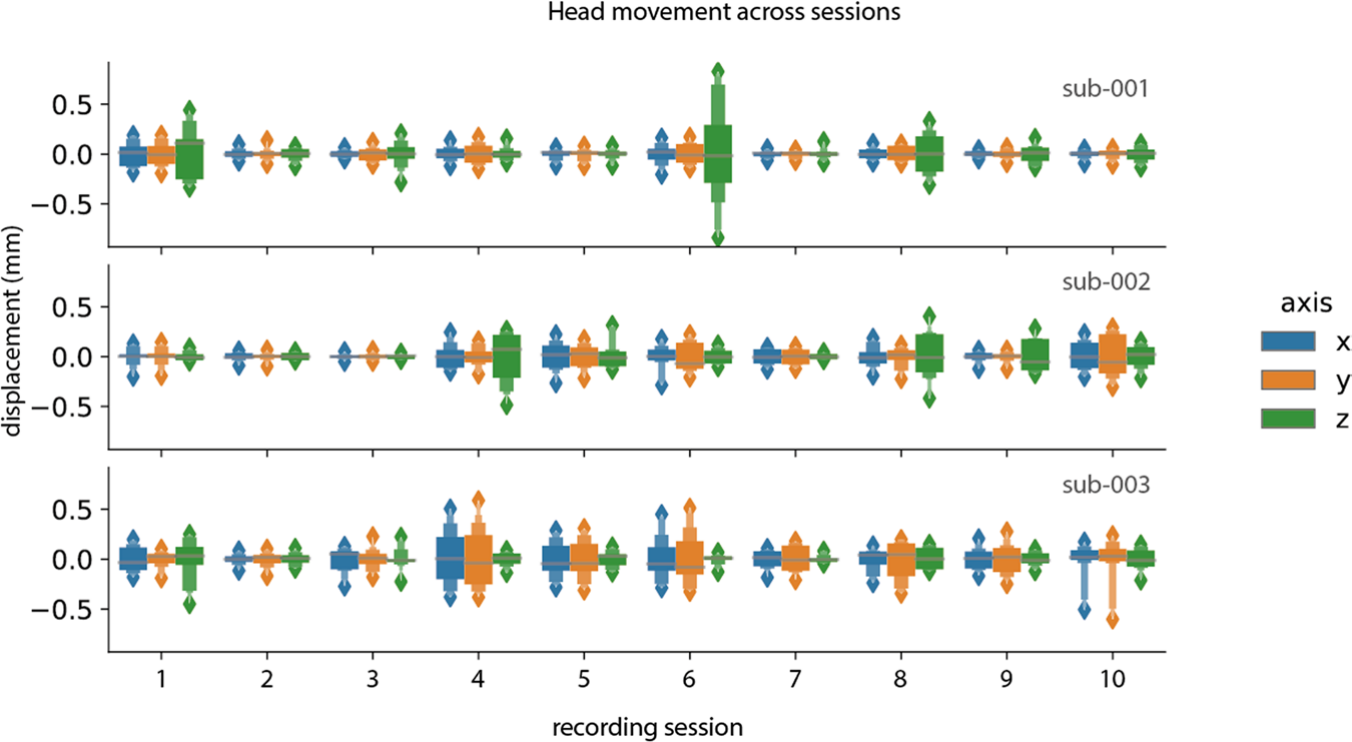
Head movement per session and per participant.

In Fig. 6, we show head movement data in a previously published dataset without headcast^70^ and the current dataset. The dataset without headcast shows clear movement displacement in all directions within the reported limits of 5 mm. In the current dataset, the head movement was maintained below 1 millimeter throughout the recordings. This holds across all three participants (Fig. 7).

### Evoked responses

To provide an impression of the MEG data in the dataset, we perform a basic analysis of auditory evoked responses^71^ per participant and every session in the dataset.

#### Preprocessing

Prior to preprocessing, we first demeaned the raw data. Next, we applied a band-pass filter (hamming-windowed sync FIR filter via fast fourier transform: cutoff (−6 dB) 1 Hz and 40 Hz, transition width 2.0 Hz, stopband 0–0.0 Hz, passband 2.0–39.0 Hz, stopband 41.0–600 Hz, max. passband deviation 0.0022 (0.22%), stopband attenuation −53 dB). We then applied notch filtering (Butterworh IIR) at the bandwidth of 49–51, 99–101, and 149–151 Hz to remove the potential line noise artifacts. Artifacts related to muscle contraction and squidjumps were identified and removed using a semi-automatic artifact rejection procedure (http://www.fieldtriptoolbox.org/tutorial/automatic_artifact_rejection). The data were then downsampled to 150 Hz. MEG components reflecting eye-blinks were estimated using the FastICA algorithm (https://research.ics.aalto.fi/ica/fastica/) as implemented in Fieldtrip functionalities). ICA was performed separately per each run (‘story subsection’) in every recording session. Relevant components were identified based on their topography and time-courses and removed from the data.

#### Source reconstruction

##### Cortical sheet reconstruction

To localize fiducial coils (the nasion, left and right ear) on participants’ MRI images in the MNI coordinate space, we used the position information of where the digitized fiducials were placed on the headcasts in the CTF space (see Section ‘MRI-MEG coregistration’). After co-registration, we used the Brain Extraction Tool^72^ from the FSL command-line library (v5.0.9.)^73^ to delete the non-brain tissue (skull striping) from the whole head. To obtain a description of individual participant’s cortical sheet, we performed cortical surface reconstruction with the Freesurfer image analysis suite, which is documented and freely available for download online (http://surfer.nmr.mgh.harvard.edu/), using the surface-based stream implemented in the ‘recon_all’ command-line tool. The post-processing of the reconstructed cortical surfaces was performed using the Connectome Workbench ‘wb_command’ command-line tools (v1.1.1; https://www.humanconnectome.org/software/workbench-command).

##### Beamformer

The cortical sheet reconstruction procedure described above resulted in a description of individual participant’s locations of potential neural sources along the cortical sheet (source model) with 7,842 source locations per hemisphere. We used a single-shell spherical volume conduction model based on a realistic shaped surface of the inside of the skull^74^ to compute the forward projection matrices (leadfields). We used a common leadfield for estimating session-specific beamformer weights. To estimate MEG source time series, we used linearly constrained minimum variance (LCMV) spatial filtering^75^ deployed with ‘ft_sourceanalysis’ routine. Source reconstruction was performed separately per each recording session. Data of all runs in a session were used to compute the data covariance matrix for beamformer computation. Source parcels (grouping of source points into brain areas or parcels) were created using a refined version of the Conte69 atlas (brainvis.wustl.edu/wiki/index.php//Caret:Atlases/Conte69?_Atlas), which provides a parcellation of the neocortical surface based on Brodmann’s cytoarchitectonic atlas. The original atlas, consisting of 41 labeled parcels per hemisphere, was further refined to obtain 187 equisized parcels, observing the parcel boundaries of the original atlas.

#### Event-related fields

**Fig. 8:**
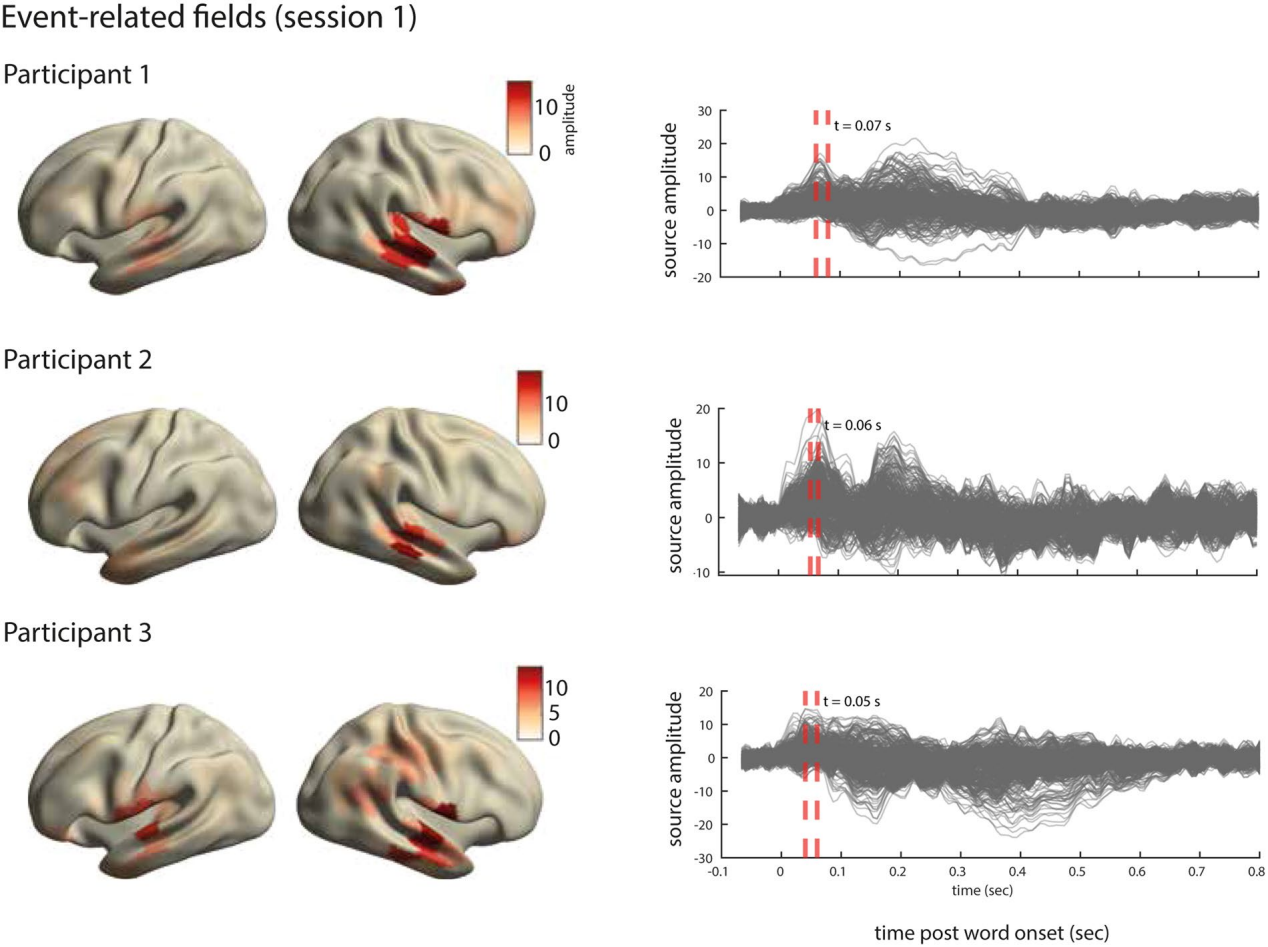
Analysis of evoked responses for one session in the dataset. Right. Line plots show the averaged source time courses (ERFs) for all brain parcels (each line represents a brain parcel). Time point 0 on the time axis indicates the word onset. Left. The source topographies show the distribution of activations designated by the orange dashed line in the ERF time courses on the right. We selected the time points that approximately correspond to the peak activation of the earliest component post word-onset.

The outcome of the preprocessing and source-reconstruction steps are MEG time series for 370 brain parcels in the left and right hemispheres. To obtain the average event-related field (ERF) for each source parcel, we first concatenated all runs within each session. We then epoched the MEG time-series in time windows starting from 100 ms prior to word-onset and extending to 500 msec post word-onset. We then compute the average across the epochs. This results, for each participant and each recording session, in an average representation of the word-onset evoked signal for every source parcel. The results for session 1 of each participant are displayed in Fig. 8.

**Fig. 9:**
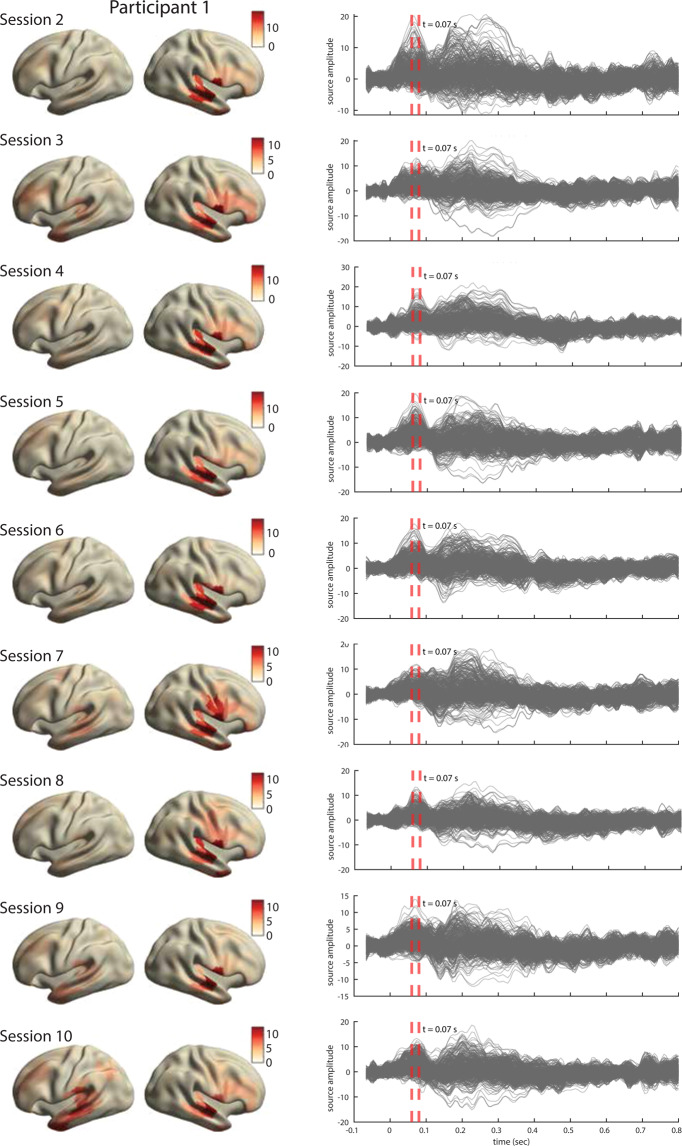
Analysis of evoked responses for participant 1, sessions 2 through 10. **Right**. Line plots show the averaged source time courses (ERFs) for all brain parcels (each line represents a brain parcel). Time point 0 on the time axis indicates the word onset. **Left**. The source topographies show the distribution of average activation within the interval designated by the orange dashed line in the ERF time courses on the right. We selected the time points that approximately correspond to the peak activation of the earliest component post word-onset.

**Fig. 10:**
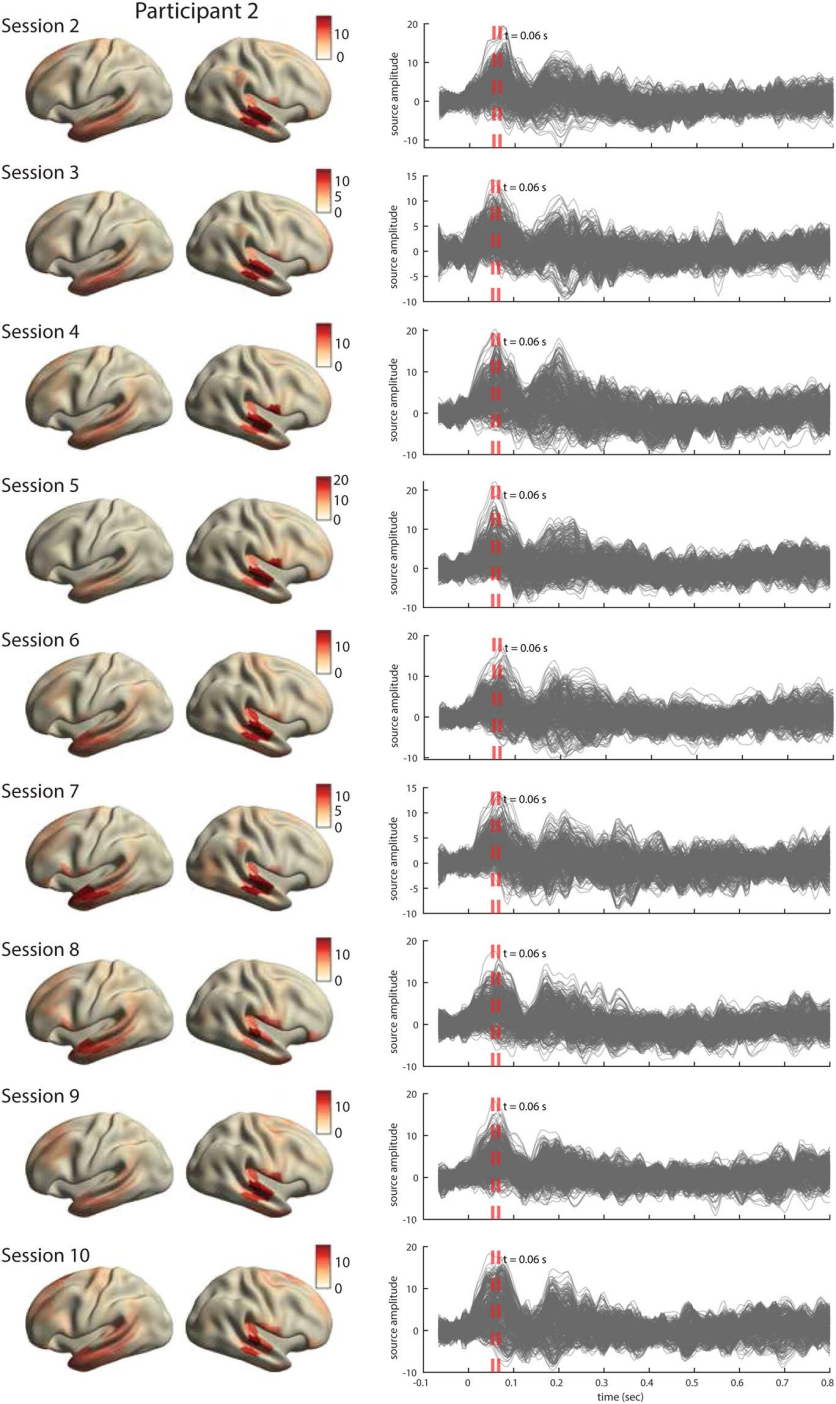
Analysis of evoked responses for participant 2, sessions 2 through 10. Right. Line plots show the averaged source time courses (ERFs) for all brain parcels (each line represents a brain parcel). Time point 0 on the time axis indicates the word onset. Left. The source topographies show the distribution of average activation within the interval designated by the orange dashed line in the ERF time courses on the right. We selected the time points that approximately correspond to the peak activation of the earliest component post word-onset.

**Fig. 11:**
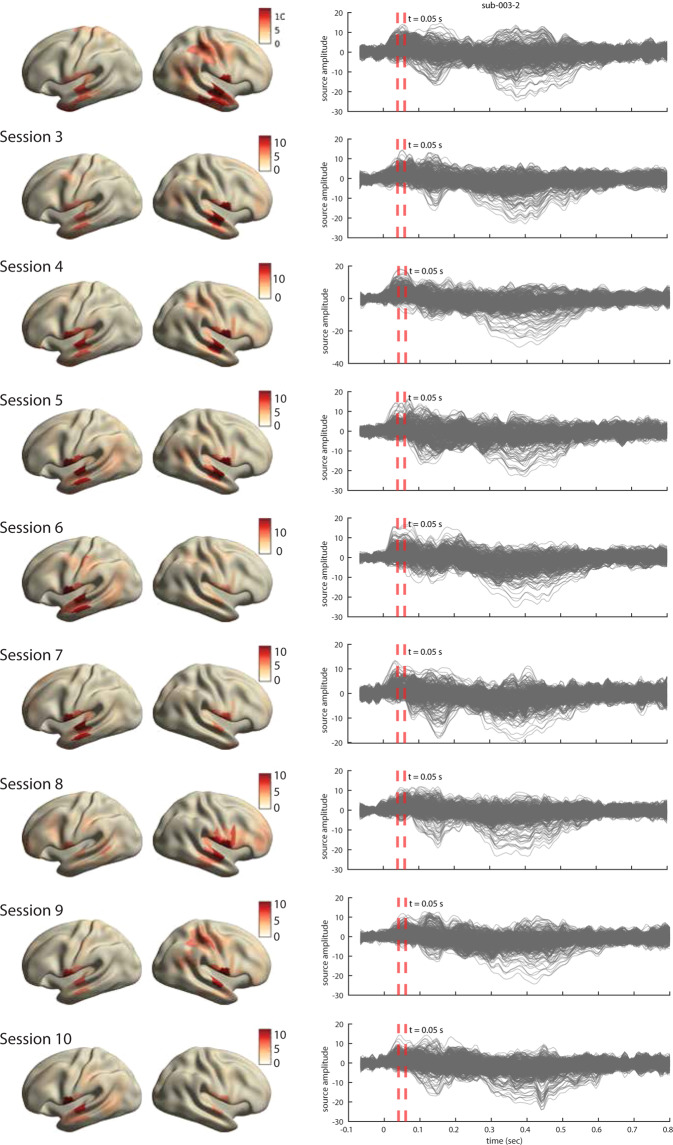
Analysis of evoked responses for participant 3, sessions 2 through 10. Right. Line plots show the averaged source time courses (ERFs) for all brain parcels (each line represents a brain parcel). Time point 0 on the time axis indicates a word onset. Left. The source topographies show the distribution of average activation within the interval designated by the orange dashed line in the ERF time courses on the right. We selected the time points that approximately correspond to the peak activation of the earliest component post word-onset.

For all three participants, the ERFs show the expected temporal profile with early peaks at approximately 50 msec post word-onsets (Fig. 8, right). The inspection of source topographies at selected latencies (orange dashed lines in the right-hand side panels) shows clear focal topographies with peak activations localized in the primary auditory cortex in the superior temporal gyrus. The patterns are right-lateralized for participants 1 and 2 whereas they show a more bilateral pattern in participant 3. Such inter-individual variability in brain activation patterns in auditory language comprehension has been reported in MEG previously^76^. The ERFs show a broader range of frequencies over time than is perhaps typically shown in an ERP/ERF analysis. It should be noted that we use a rather broad bandpass filter (0.5-40 Hz) compared to other reports (e.g. Broderick *et al*.^77^ filter at 1-8 Hz). Finally and importantly, the results are robust and consistent across all ten sessions in each participant. The results for other sessions for all participants are shown in Figs. 9–11.

### Repeat reliability

After each run, we recorded two short 30 seconds long snippets repeated one after another (see Fig. 2). This repeated design across runs, sessions and participants allows for estimation of signal reliability that can inform main analyses of story runs. To do a basic quality analysis, we performed a canonical correlation analysis (CCA) where we train a linear model ${f}_{k}$ on a pair of repeated runs, ${{\bf{Y}}}^{train}=[{{\bf{Y}}}_{r}^{1},{{\bf{Y}}}_{r}^{2}]$. The runs in the pair *r* are taken either from the same session (withing session condition) or from two different sessions (across session condition). The model ${f}_{r}$ trained on a pair of repeats *r* learns a set of linear weights ${\widehat{{\bf{W}}}}_{r}$ that maximize the correlation between the two repeats in the pair. We then evaluate the model ${f}_{r}$ on a pair of held out repeats $p;p\ne r$ not used in the training step. Specifically, model performance is quantified by computing the correlation between the data segments in the pair *p* after the model is applied: $corr({f}_{r}({{\bf{Y}}}_{p}^{2}),{f}_{r}({{\bf{Y}}}_{p}^{1}))$. The rationale behind this evaluation procedure is that the presence of any shared brain features uncovered by the CCA model ${f}_{k}$ would lead to non-zero correlation when applied to unseen data. We compare the model trained on repeats ${f}_{k}$ against a baseline CCA model trained on pairs of 30-second snippets selected at random from the *story* runs (i.e. each 30-second snippet was recorded to different inputs). Put differently, the brain signal in the *repeated* segments condition (more precisely, the canonical variates) is predominantly expected to be driven by the shared components due to input repetition, whereas it is expect to contain less of the shared components across randomly selected paired segments.

We show (see Table 1) that there was a main effect of ‘segment type’, that is, canonical variates obtained from *repeated segments* were significantly different from canonical variates obtained from *randomly sampled segments* (sampled either within or across sessions). However, there was no effect of ‘session’, that is, comparing canonical variates based on segments sampled from within or across sessions were not significantly different (regardless of whether these were randomly selected story segments or repeated segments). This confirms that neural responses to repeated segments have a larger degree of shared signal component due to input repetition and, importantly, that this is consistent across sessions in all three participants.

## Usage Notes

### Interpretation of behavioral log files (‘_beh.txt’)

Each row in the tab-separated value file corresponds to a run in the session and contains responses to the behavioral questions that were answered after each run. Description of each variable is given in Table 2.

**Table 2:** Description of variables in *beh.txt files.

Variable name	Description
date	date when the session recording took place
time	date and time when the session recording took place
story nr.	session number, an integer representing the story index that was listened to in that recording session
session nr.	run number, an integer representing the run index in that session
response	recorded response (button box) to the multiple choice question (possible values: A, B or C)
correct	the correct response to the multiple choice question (possible values: A, B or C)
ishit	whether or not the given response matches the true response (can be 1 or 0)
absorption_score	the response to the absorption question (possible values: 1 through 7)
density_score	the response to the information density question (possible values: 1 through 7)

### Interpretation of the ‘events.tsv’ file

The ‘events.tsv’ file logs the events read from the MEG header files. In addition, the ‘events.tsv’ file also contains the aligned events from the ‘.log’ log files which contain a record of events generated by the Presentation experimental scripts. The event onsets are adjusted for the estimated delays between the Presentation trigger sending and their recording in the CTF acquisition system. Not all triggers from the Presentation script were sent to the CTF trigger channel. For completeness, we provide the mapping between the experimentally-relevant codes in the Presentation log files and the CTF trigger channel in Table 3. An illustrative (edited) example of the ‘events.tsv’ file is shown in Table 4.

**Table 3:** Mapping between events in the experimentally relevant code in the Presentation log files and the CTF trigger channel.

trigger value	Presentation type	CTF trigger type	description
%d0	Sound	UPPT001	story onset, the start of.wav file playback
%d5	Picture	UPPT001	story offset, marked by the onset of screen indicating end of story/run
%d7	Picture	UPPT001	presentation of screen showing comprehension check
%d8	Picture	UPPT001	presentation of screen showing information density question
%d9	Picture	UPPT001	presentation of screen showing absorption question
100	Sound	UPPT001	repeated stimulus onset, the start of.wav file playback
150	Picture	UPPT001	repeated stimulus offset, marked by the onset of screen indicating end of the repetition stimulus

The trigger values are two-digit codes where the first digit codes the run index. The second digit codes the identity of the event (i.e. story onset, offset etc.).

**Table 4:** A sample of an ‘*events.tsv’ file.

	onset	duration	sample	type	value
0	37.774454	NaN	45330.344827	wav_onset	10
1	37.786092	1.077551	45344.310813	word_onset_01	sp
2	37.786092	1.077551	45344.310813	phoneme_onset_01	sp
3	38.863643	0.129705	46637.372038	word_onset_01	THE
4	38.863643	0.059864	46637.372038	phoneme_onset_01	DH
5	38.923507	0.069841	46709.208773	phoneme_onset_01	IY0
6	38.993349	0.678458	46793.018296	word_onset_01	ADVENTURES
7	38.993349	0.029932	46793.018296	phoneme_onset_01	AH0
8	39.023281	0.049887	46828.936664	phoneme_onset_01	D
9	39.073167	0.059864	46888.800609	phoneme_onset_01	V
10	39.133031	0.069841	46960.637344	phoneme_onset_01	EH1
11	39.202872	0.099773	47044.446868	phoneme_onset_01	N
12	39.302646	0.129705	47164.174759	phoneme_onset_01	CH
13	39.432351	0.069841	47319.821017	phoneme_onset_01	ER0
14	39.502192	0.169615	47403.630541	phoneme_onset_01	Z
6124	568.528583	NaN	682235.300153	wav_onset	100
6125	604.108805	NaN	724931.566321	wav_onset	100
6126	775.129892	NaN	930156.870608	wav_onset	20
6127	775.141530	0.957823	930170.836595	word_onset_02	sp
6128	775.141530	0.957823	930170.836595	phoneme_onset_02	sp
6129	776.099354	0.059864	931320.224350	word_onset_02	THE
6130	776.099354	0.029932	931320.224350	phoneme_onset_02	DH
6131	776.129286	0.029932	931356.142717	phoneme_onset_02	AH0
6132	776.159218	0.319274	931392.061085	word_onset_02	NOTE
6133	776.159218	0.139683	931392.061085	phoneme_onset_02	N
6134	776.298900	0.099773	931559.680132	phoneme_onset_02	OW1
6135	776.398673	0.079819	931679.408023	phoneme_onset_02	T
6136	776.478492	0.169615	931775.190336	word_onset_02	WAS
6137	776.478492	0.059864	931775.190336	phoneme_onset_02	W
6138	776.538356	0.029932	931847.027071	phoneme_onset_02	AH0
6139	776.568288	0.079819	931882.945438	phoneme_onset_02	Z
6140	776.648106	0.698413	931978.727751	word_onset_02	UNDATED

Each row logs one event relative to the MEG recording session onset. Time values are in seconds. For this example, we only show the first few events from session 1 and session 2 (hence, the discontinuity in row indices).

### Additional recording channels

In Table 5 we provide the information about the additional channels recorded along with the MEG data.

**Table 5:** Additional recording channels in the MEG datasets.

channel label	description
UADC003	analog input channel with audio signal
UADC004	analog input channel with audio signal
UADC005	eye-tracker, x-position
UADC006	eye-tracker, y-position
UADC007	eye-tracker, pupil diameter

**Table 6:** Literary appreciation questionnaire.

Question
I thought this was a good story
I thought this story was... Beautiful
I thought this story was... Boring
I thought this story was... Deeply moving
I thought this story was... Entertaining
I thought this story was... Funny
I thought this story was... Interesting
I thought this story was... Ominous
I thought this story was... Sad
I thought this story was... Suspenseful
I thought this story was... Tragic
I thought this story was... Witty
I thought this story was... Captivating
I thought this story was... Special
I was constantly curious about how the story would end
I thought it was fun to read this story
I want to read the story again sometimes
I thought the story was written well
I rather did not want the story to end
I would recommend this story to somebody else

## Known Exceptions and Issues

### Bad channels

The following channels in this dataset show unstable behavior due to technical issues in the lab at the time of recording for sub-003, ses-004: MRC23, MLO22, MRP5, MLC23. Researchers are advised to remove the above channels during preprocessing.

### Repeated run

Due to technical issues, the experimental script crashed during run 3 for sub-003, ses-008. We restarted the experiment at run 3. This means parts of run 3 before the experiment stopped were listened to twice and only the second iteration of run 3 is complete.

### Low-frequency artifacts

We noticed short-lived (approximately a couple of seconds in duration), but high-amplitude, slow-drift artifacts in the following runs:

- sub-002, ses-009, run 1
- sub-003, ses-004, run 4
- sub-003, ses-005, run 1
- sub-003, ses-006, run 6
- sub-003, ses-008, run 4

Depending on the research question and preprocessing steps (e.g. the use of a narrow-band filter), the presence of artifacts might not be detrimental. Otherwise, high-pass filtering with a low cut-off (e.g. 0.5 Hz) might be required to suppress these artifacts.

### Spike-like artifact in two channels (sub-003, ses-003)

In sub-003, ses-003, channel MRP57 shows an uncharacteristic regular (approximately every 10 seconds or longer), but short-lived impulse-like events. We could not determine the origin of this artifact. In our experience, the artifact can be detected using established blind-source estimation techniques (e.g. independent component analysis) and removed from the data. Given its sparse temporal nature, we estimate it being unlikely that this artifact can significantly affect the quality of the dataset, but warrants consideration nevertheless.

### Exceptions in appreciation measurements

For sub-001, ses-001, part of appreciation questionnaires were corrected offline. This corrected behavioral response documented in the behavioral log file with the suffix ‘_offline’. The reasons for these exceptions were due to inadvertent wrong button press and were reported to the responsible researcher immediately after the session ended. The file containing the entry with the correct response was created in order to keep the old and new response logged explicitly.

The appreciation measurements for sub-003, ses-008 are recorded in two files (ses-008A and ses-00B) due to the experimental script crashing (see Section ‘Repeated Run’).

## Supplementary information

Supplementary Table 1

A 10-hour within-participant magnetoencephalography narrative dataset to test models of naturalistic language comprehension: Supplementary information

## Data Availability

The code is available as part of the data collection at the data repository of the Donders Institute for Brain, Cognition and Behaviour.

## References

[1] Puchner, M. *The written world: the power of stories to shape people, history, civilization* first edition edn (Random House, New York, 2017).

[2] Bruner, J. The narrative construction of reality. *Critical Inquiry* **18**, 1–21 (1991). Publisher: The University of Chicago Press.

[3] White H (1980). The value of narrativity in the representation of reality. Critical Inquiry.

[4] Hasson U, Honey CJ (2012). Future trends in neuroimaging: neural processes as expressed within real-life contexts. NeuroImage.

[5] Willems, R. M. (ed.) *Cognitive neuroscience of natural language use* (Cambridge University Press, Cambridge, United Kingdom, 2015).

[6] Brennan JR (2016). Naturalistic sentence comprehension in the brain: naturalistic comprehension. Language and Linguistics Compass.

[7] Matusz PJ, Dikker S, Huth AG, Perrodin C (2018). Are we ready for real-world neuroscience?. Journal of Cognitive Neuroscience.

[8] Kandylaki KD, Bornkessel-Schlesewsky I (2019). From story comprehension to the neurobiology of language. Language, Cognition and Neuroscience.

[9] Hamilton LS, Huth AG (2018). The revolution will not be controlled: natural stimuli in speech neuroscience. Language, Cognition and Neuroscience.

[10] Nastase SA, Goldstein A, Hasson U (2020). Keep it real: rethinking the primacy of experimental control in cognitive neuroscience. NeuroImage.

[11] Willems RM, Nastase SA, Milivojevic B (2020). Narratives for neuroscience. Trends in Neurosciences.

[12] Wehbe L (2014). Simultaneously uncovering the patterns of brain regions involved in different story reading subprocesses. PLoS ONE.

[13] Armeni K, Willems RM, Frank SL (2017). Probabilistic language models in cognitive neuroscience: Promises and pitfalls. Neuroscience & Biobehavioral Reviews.

[14] LeCun Y, Bengio Y, Hinton G (2015). Deep learning. Nature.

[15] Sejnowski, T. J. The unreasonable effectiveness of deep learning in artificial intelligence. *Proceedings of the National Academy of Sciences* 201907373, 10.1073/pnas.1907373117 (2020). 10.1073/pnas.1907373117 PMC7720171 31992643

[16] Goldberg, Y. *Neural network methods for natural language processing*. No. 37 in Synthesis lectures on human language technologies (Morgan & Claypool Publishers, San Rafael, 2017). OCLC: 990794614.

[17] Bengio Y, Ducharme R, Vincent P, Jauvin C (2003). A neural probabilistic language model. Journal of Machine Learning Research.

[18] Mikolov, T., Karafiát, M., Burget, L., Cernocký, J. & Khudanpur, S. Recurrent neural network based language model. In *INTERSPEECH 2010, 11th Annual Conference of the International Speech Communication Association, Makuhari, Chiba, Japan, September 26-30*, 2010, 1045–1048 (2010).

[19] Mikolov, T., Sutskever, I., Chen, K., Corrado, G. & Dean, J. Distributed representations of words and phrases and their compositionality. In *Proceedings of the 26th International Conference on Neural Information Processing Systems - Volume 2*, NIPS’13, 3111–3119 (Curran Associates Inc., Red Hook, NY, USA, 2013). Event-place: Lake Tahoe, Nevada.

[20] Pennington, J., Socher, R. & Manning, C. Glove: Global vectors for word representation. In *Proceedings of the 2014 Conference on Empirical Methods in Natural Language Processing (EMNLP)*, 1532–1543, 10.3115/v1/D14-1162 (Association for Computational Linguistics, Doha, Qatar, 2014).

[21] Devlin, J., Chang, M.-W., Lee, K. & Toutanova, K. BERT: Pre-training of deep bidirectional transformers for language understanding. In *Proceedings of the 2019 Conference of the North American Chapter of the Association for Computational Linguistics: Human Language Technologies, Volume 1 (Long and Short Papers)*, 4171–4186, 10.18653/v1/N19-1423 (Association for Computational Linguistics, Minneapolis, Minnesota, 2019).

[22] Radford, A. *et al*. Language models are unsupervised multitask learners (2019).

[23] Brown, T. *et al*. Language models are few-shot learners. In Larochelle, H., Ranzato, M., Hadsell, R., Balcan, M. F. & Lin, H. (eds.) *Advances in Neural Information Processing Systems*, **vol. 33**, 1877–1901 (Curran Associates, Inc., 2020).

[24] Krizhevsky, A., Sutskever, I. & Hinton, G. E. ImageNet classification with deep convolutional neural networks. In Pereira, F., Burges, C. J. C., Bottou, L. & Weinberger, K. Q. (eds.) *Advances in Neural Information Processing Systems 25*, 1097–1105 (Curran Associates, Inc., 2012). Krizhevsky_imagenet_2012.

[25] Kriegeskorte N (2015). Deep neural networks: A new framework for modeling biological vision and brain information processing. Annual Review of Vision Science.

[26] Güçlü U, Gerven MAJV (2015). Deep neural networks reveal a gradient in the complexity of neural representations across the ventral stream. Journal of Neuroscience.

[27] Yamins DL, DiCarlo JJ (2016). Using goal-driven deep learning models to understand sensory cortex. Nature Neuroscience.

[28] Alishahi A, Chrupała G, Linzen T (2019). Analyzing and interpreting neural networks for NLP: A report on the first BlackboxNLP workshop. Natural Language Engineering.

[29] Giulianelli, M., Harding, J., Mohnert, F., Hupkes, D. & Zuidema, W. Under the hood: Using diagnostic classifiers to investigate and improve how language models track agreement information. In *Proceedings of the 2018 EMNLP Workshop BlackboxNLP: Analyzing and Interpreting Neural Networks for NLP*, 240–248, 10.18653/v1/W18-5426 (Association for Computational Linguistics, Brussels, Belgium, 2018).

[30] Manning, C. D., Clark, K., Hewitt, J., Khandelwal, U. & Levy, O. Emergent linguistic structure in artificial neural networks trained by self-supervision. *Proceedings of the National Academy of Sciences*10.1073/pnas.1907367117 (2020). Publisher: National Academy of Sciences Section: Physical Sciences. 10.1073/pnas.1907367117 PMC7720155 32493748

[31] Mitchell TM (2008). Predicting human brain activity associated with the meanings of nouns. Science.

[32] Pereira F (2018). Toward a universal decoder of linguistic meaning from brain activation. Nature Communications.

[33] Gauthier, J. & Levy, R. Linking artificial and human neural representations of language. In *Proceedings of the 2019 Conference on Empirical Methods in Natural Language Processing and the 9th International Joint Conference on Natural Language Processing (EMNLP-IJCNLP)*, 529–539, 10.18653/v1/D19-1050 (Association for Computational Linguistics, Hong Kong, China, 2019).

[34] Wehbe, L., Vaswani, A., Knight, K. & Mitchell, T. M. Aligning context-based statistical models of language with brain activity during reading. In *EMNLP*, 233–243 (ACL, 2014).

[35] Huth AG, Heer WAD, Griffiths TL, Theunissen FE, Gallant JL (2016). Natural speech reveals the semantic maps that tile human cerebral cortex. Nature.

[36] Berezutskaya J, Freudenburg ZV, Güçlü U, Gerven MAJV, Ramsey NF (2017). Neural tuning to low-level features of speech throughout the perisylvian cortex. Journal of Neuroscience.

[37] Caucheteux, C. & King, J.-R. Language processing in brains and deep neural networks: computational convergence and its limits. *bioRxiv* 2020.07.03.186288, 10.1101/2020.07.03.186288, Publisher: Cold Spring Harbor Laboratory Section: New Results (2020).

[38] Cichy RM, Kaiser D (2019). Deep neural networks as scientific models. Trends in Cognitive Sciences.

[39] Kriegeskorte N, Golan T (2019). Neural network models and deep learning. Current Biology.

[40] Marblestone, A. H., Wayne, G. & Kording, K. P. Toward an integration of deep learning and neuroscience. *Frontiers in Computational Neuroscience* **10**, 10.3389/fncom.2016.00094 (2016). 10.3389/fncom.2016.00094 PMC5021692 27683554

[41] Richards BA (2019). A deep learning framework for neuroscience. Nature Neuroscience.

[42] Fong RC, Scheirer WJ, Cox DD (2018). Using human brain activity to guide machine learning. Scientific Reports.

[43] Sinz FH, Pitkow X, Reimer J, Bethge M, Tolias AS (2019). Engineering a less artificial intelligence. Neuron.

[44] Hassabis D, Kumaran D, Summerfield C, Botvinick M (2017). Neuroscience-inspired artificial intelligence. Neuron.

[45] Geman S, Bienenstock E, Doursat R (1992). Neural networks and the bias/variance dilemma. Neural Computation.

[46] Schoffelen J-M (2019). A 204-subject multimodal neuroimaging dataset to study language processing. Scientific Data.

[47] Nastase SA (2021). The “Narratives” fMRI dataset for evaluating models of naturalistic language comprehension. Scientific Data.

[48] Seeliger, K. *et al*. Convolutional neural network-based encoding and decoding of visual object recognition in space and time. *NeuroImage*10.1016/j.neuroimage.2017.07.018 (2017). 10.1016/j.neuroimage.2017.07.018 28723578

[49] Anumanchipalli GK, Chartier J, Chang EF (2019). Speech synthesis from neural decoding of spoken sentences. Nature.

[50] Makin JG, Moses DA, Chang EF (2020). Machine translation of cortical activity to text with an encoder–decoder framework. Nature Neuroscience.

[51] Güçlü, U. & van Gerven, M. A. J. Modeling the dynamics of human brain activity with recurrent neural networks. *Frontiers in Computational Neuroscience* **11**, 10.3389/fncom.2017.00007 (2017). 10.3389/fncom.2017.00007 PMC5299026 28232797

[52] Smith, P. L. & Little, D. R. Small is beautiful: In defense of the small-N design. *Psychonomic Bulletin & Review* 1–19, 10.3758/s13423-018-1451-8 (2018). 10.3758/s13423-018-1451-8 PMC6267527 29557067

[53] Baillet S, Mosher JC, Leahy RM (2001). Electromagnetic brain mapping. IEEE Signal Processing Magazine.

[54] Lopes da Silva F (2013). EEG and MEG: Relevance to neuroscience. Neuron.

[55] Hämäläinen M, Hari R, Ilmoniemi RJ, Knuutila J, Lounasmaa OV (1993). Magnetoencephalography—theory, instrumentation, and applications to noninvasive studies of the working human brain. Reviews of Modern Physics.

[56] Baillet S (2017). Magnetoencephalography for brain electrophysiology and imaging. Nature Neuroscience.

[57] Holdgraf, C. R. *et al*. Encoding and decoding models in cognitive electrophysiology. *Frontiers in Systems Neuroscience* **11**, 10.3389/fnsys.2017.00061 (2017). 10.3389/fnsys.2017.00061 PMC5623038 29018336

[58] Gross J (2013). Good practice for conducting and reporting MEG research. NeuroImage.

[59] Pernet, C. R. *et al*. Best practices in data analysis and sharing in neuroimaging using MEEG. *preprint, Open Science Framework*10.31219/osf.io/a8dhx (2018).

[60] Strauber CB, Ali LR, Fujioka T, Thille C, McCandliss BD (2021). Replicability of neural responses to speech accent is driven by study design and analytical parameters. Scientific Reports.

[61] Yuan J, Liberman M (2008). Speaker identification on the SCOTUS corpus. The Journal of the Acoustical Society of America.

[62] Mak M, Willems RM (2019). Mental simulation during literary reading: Individual differences revealed with eye-tracking. Language, Cognition and Neuroscience.

[63] Knoop CA, Wagner V, Jacobsen T, Menninghaus W (2016). Mapping the aesthetic space of literature “from below”. Poetics.

[64] Kuijpers MM, Hakemulder F, Tan ES, Doicaru MM (2014). Exploring absorbing reading experiences: Developing and validating a self-report scale to measure story world absorption. Scientific Study of Literature.

[65] Meyer SS (2017). Flexible head-casts for high spatial precision MEG. Journal of Neuroscience Methods.

[66] Stolk A, Todorovic A, Schoffelen J-M, Oostenveld R (2013). Online and offline tools for head movement compensation in MEG. NeuroImage.

[67] Armeni K, Güçlü U, van Gerven M, Schoffelen J-M (2022). Donders Data Repository.

[68] Niso, G. *et al*. MEG-BIDS, the brain imaging data structure extended to magnetoencephalography. *Scientific Data* **5**, 10.1038/sdata.2018.110 (2018). 10.1038/sdata.2018.110 PMC6007085 29917016

[69] Oostenveld, R., Fries, P., Maris, E. & Schoffelen, J.-M. FieldTrip: Open Source Software for Advanced Analysis of MEG, EEG, and Invasive Electrophysiological Data. *Computational Intelligence and Neuroscience*10.1155/2011/156869 (2011). 10.1155/2011/156869 PMC3021840 21253357

[70] Armeni, K., Willems, R. M., van den Bosch, A. & Schoffelen, J.-M. Frequency-specific brain dynamics related to prediction during language comprehension. *NeuroImage*10.1016/j.neuroimage.2019.04.083 (2019). 10.1016/j.neuroimage.2019.04.083 31100432

[71] Luck, S. J. *An introduction to the event-related potential technique* (The MIT Press, Cambridge, Massachusetts, 2014), second edn.

[72] Smith SM (2002). Fast robust automated brain extraction. Human Brain Mapping.

[73] Jenkinson M, Beckmann CF, Behrens TE, Woolrich MW, Smith SM (2012). FSL. NeuroImage.

[74] Nolte G (2003). The magnetic lead field theorem in the quasi-static approximation and its use for magnetoencephalography forward calculation in realistic volume conductors. Physics in Medicine and Biology.

[75] Veen BDV, Drongelen WV, Yuchtman M, Suzuki A (1997). Localization of brain electrical activity via linearly constrained minimum variance spatial filtering. IEEE Transactions on Biomedical Engineering.

[76] Lam NHL, Hultén A, Hagoort P, Schoffelen J-M (2018). Robust neuronal oscillatory entrainment to speech displays individual variation in lateralisation. Language, Cognition and Neuroscience.

[77] Broderick, M. P., Anderson, A. J., Liberto, G. M. D., Crosse, M. J. & Lalor, E. C. Electrophysiological correlates of semantic dissimilarity reflect the comprehension of natural, narrative speech. *Current Biology* **0**, 10.1016/j.cub.2018.01.080 (2018). 10.1016/j.cub.2018.01.080 29478856

