# Supplementary material for: A 10-hour within-participant magnetoencephalography narrative dataset to test models of language comprehension
Source: Sci Data. 2022 Jun 8;9:278. doi: 10.1038/s41597-022-01382-7 (PMC9177538; doi:10.1038/s41597-022-01382-7)
Supplement: Supplementary file 2 — A 10-hour within-participant magnetoencephalography narrative dataset to test models of naturalistic language comprehension: Supplementary information [file 41597_2022_1382_MOESM2_ESM.pdf]

A 10-hour within-participant magnetoencephalography narrative dataset to test models of naturalistic language comprehension: Supplementary information

Table 1: Stimulus information

session	run	audio_filename	duration	txt_filename
ses-001	1	01_1.wav	07:56	01_1.txt
ses-001	2	01_2.wav	05:41	01_2.txt
ses-001	3	01_3.wav	09:50	01_3.txt
ses-001	4	01_4.wav	08:27	01_4.txt
ses-001	5	01_5.wav	07:42	01_5.txt
ses-001	6	01_6.wav	07:49	01_6.txt
ses-001	7	01_7.wav	07:22	01_7.txt
ses-002	1	02_1.wav	07:34	02_1.txt
ses-002	2	02_2.wav	08:52	02_2.txt
ses-002	3	02_3.wav	09:03	02_3.txt
ses-002	4	02_4.wav	07:44	02_4.txt
ses-002	5	02_5.wav	07:25	02_5.txt
ses-002	6	02_6.wav	09:04	02_6.txt
ses-002	7	02_7.wav	08:51	02_7.txt
ses-003	1	03_1.wav	06:15	03_1.txt
ses-003	2	03_2.wav	04:59	03_2.txt
ses-003	3	03_3.wav	10:27	03_3.txt
ses-003	4	03_4.wav	07:26	03_4.txt
ses-003	5	03_5.wav	05:46	03_5.txt
ses-003	6	03_6.wav	07:42	03_6.txt
ses-004	1	04_1.wav	07:43	04_1.txt

Continued on next page

Table 1: Stimulus information

session	run	audio_filename	duration	txt_filename
ses-004	2	04_2.wav	05:27	04_2.txt
ses-004	3	04_3.wav	06:55	04_3.txt
ses-004	4	04_4.wav	06:54	04_4.txt
ses-004	5	04_5.wav	09:34	04_5.txt
ses-004	6	04_6.wav	07:29	04_6.txt
ses-004	7	04_7.wav	08:37	04_7.txt
ses-004	8	04_8.wav	07:58	04_8.txt
ses-005	1	05_1.wav	06:56	05_1.txt
ses-005	2	05_2.wav	09:05	05_2.txt
ses-005	3	05_3.wav	05:48	05_3.txt
ses-005	4	05_4.wav	06:34	05_4.txt
ses-005	5	05_5.wav	11:03	05_5.txt
ses-005	6	05_6.wav	08:09	05_6.txt
ses-006	1	06_1.wav	10:25	06_1.txt
ses-006	2	06_2.wav	05:47	06_2.txt
ses-006	3	06_3.wav	09:16	06_3.txt
ses-006	4	06_4.wav	05:43	06_4.txt
ses-006	5	06_5.wav	08:36	06_5.txt
ses-006	6	06_6.wav	08:08	06_6.txt
ses-006	7	06_7.wav	09:37	06_7.txt
ses-007	1	07_1.wav	07:01	07_1.txt
ses-007	2	07_2.wav	09:51	07_2.txt
ses-007	3	07_3.wav	09:41	07_3.txt
ses-007	4	07_4.wav	08:28	07_4.txt
ses-007	5	07_5.wav	06:51	07_5.txt
ses-007	6	07_6.wav	06:53	07_6.txt
ses-008	1	08_1.wav	07:23	08_1.txt
ses-008	2	08_2.wav	08:31	08_2.txt
ses-008	3	08_3.wav	08:46	08_3.txt
ses-008	4	08_4.wav	06:21	08_4.txt
ses-008	5	08_5.wav	08:51	08_5.txt
ses-008	6	08_6.wav	08:14	08_6.txt
ses-008	7	08_7.wav	12:00	08_7.txt
ses-009	1	09_1.wav	08:35	09_1.txt

Continued on next page

Table 1: Stimulus information

session	run	audio_filename	duration	txt_filename
ses-009	2	09_2.wav	11:14	09_2.txt
ses-009	3	09_3.wav	08:21	09_3.txt
ses-009	4	09_4.wav	06:41	09_4.txt
ses-009	5	09_5.wav	05:53	09_5.txt
ses-009	6	09_6.wav	08:45	09_6.txt
ses-010	1	10_1.wav	09:31	10_1.txt
ses-010	2	10_2.wav	06:29	10_2.txt
ses-010	3	10_3.wav	10:28	10_3.txt
ses-010	4	10_4.wav	07:25	10_4.txt
ses-010	5	10_5.wav	10:59	10_5.txt
ses-010	6	10_6.wav	05:54	10_6.txt
